# Prognostic factors and treatment outcomes in EGFR-mutated NSCLC with malignant pleural effusion: focus on intrathoracic chemotherapy and EGFR-TKI therapy

**DOI:** 10.3389/fonc.2025.1667197

**Published:** 2025-09-23

**Authors:** Zhuohao Huang, Jinmei Li, Haiyin Ye, Zhong Huang, Yongcun Wang, Yuliu Xie, Xiaobi Huang, Zhen Cheng, Yuting Chen, Chang Xiao, Mingchun Li, Wenmei Su

**Affiliations:** ^1^ Department of Pulmonary Oncology, Guangdong Provincial Key Laboratory of Autophagy and Major Chronic Non-Communicable Diseases, Zhanjiang Key Laboratory of Tumor Microenvironment and Organoid Research, Affiliated Hospital of Guangdong Medical University, Zhanjiang, China; ^2^ Department of Oncology, First Affiliated Hospital of Gannan Medical University, Gannan Medical University, Ganzhou, China

**Keywords:** non-small cell lung cancer (NSCLC), malignant pleural effusion (MPE), intrathoracic chemotherapy (ICT), epidermal growth factor receptor (EGFR), tyrosine kinase inhibitors (TKIs)

## Abstract

**Objective:**

In treatment-naïve patients with EGFR-mutated non-small-cell lung cancer (NSCLC) complicated by malignant pleural effusion (MPE), we first investigated whether the addition of intrathoracic chemotherapy (ICT) to first-line EGFR tyrosine-kinase inhibitors (EGFR-TKIs) confers superior therapeutic efficacy or survival outcomes compared with EGFR-TKI monotherapy. Subsequently, multivariable analyses were performed to identify independent prognostic determinants across the entire cohort, thereby informing individualized treatment selection.

**Methods:**

A retrospective analysis was performed, ultimately including 169 individuals diagnosed with stage IVA-IVB NSCLC who tested positive for EGFR mutations and exhibited malignant pleural effusion at initial presentation. All patients underwent either first-line EGFR-TKI monotherapy or a combination of intrathoracic chemotherapy with EGFR-TKIs. Patients were grouped according to receipt of EGFR-TKIs with or without concomitant ICT and by pertinent clinical characteristics. Kaplan-Meier survival analysis and Cox proportional hazards regression models were utilized to evaluate survival outcomes and potential influencing factors. The study’s objective was to determine the differential impact of intrathoracic chemotherapy plus EGFR-TKIs versus EGFR-TKIs alone on therapeutic efficacy and survival, while concurrently elucidating the independent prognostic relevance of clinical characteristics in EGFR-mutated NSCLC patients presenting with malignant pleural effusion, thereby guiding treatment prioritization.

**Results:**

Among patients with stage IVA-IVB NSCLC who were EGFR mutation-positive and presented with malignant pleural effusion at initial diagnosis, a comparative analysis showed no statistically significant differences in median progression-free survival (mPFS) (18.2 months vs. 15.0 months, Log Rank p = 0.07) and median overall survival (mOS) (29.2 months vs. 30.6 months, Log Rank p = 0.09) between EGFR-TKI monotherapy and the combination of thoracic perfusion chemotherapy with EGFR-TKIs. Further univariate and multivariate analyses indicated that the combination of EGFR-TKIs and ICT did not significantly impact PFS or OS. However, the use of third-generation EGFR-TKIs and the presence of exon 19 deletions independently predicted longer PFS, while ECOG performance status > 1, the presence of compound mutations, and liver metastasis predicted shorter OS.

**Conclusion:**

Despite our study failing to demonstrate superior efficacy or survival benefits of ICT combined with EGFR-TKIs compared to EGFR-TKI monotherapy, considering that international clinical guidelines recommend pleural drainage as a standard approach for managing MPE and the significant efficacy of third-generation EGFR-TKIs observed in our study for treating EGFR mutation-positive lung cancer patients with MPE, we speculate that the combination of third-generation EGFR-TKIs and pleural drainage may be a more rational treatment option for this patient population. Future studies are needed to further validate this hypothesis.

## 1 Background

Non-small cell lung cancer (NSCLC) is a prominent contributing factor to cancer-related mortality globally, with high incidence and fatality rates worldwide (1). Among those diagnosed with NSCLC, mutations in the epidermal growth factor receptor (EGFR) represent one of the most common driving gene alterations, especially within Asian populations (2). Individuals with NSCLC characterized by EGFR mutations typically respond positively to therapies involving EGFR tyrosine kinase inhibitors (TKIs). Nonetheless, as the disease advances, a significant number of patients encounter resistance, resulting in disease relapse and the spread of metastases (3). Additionally, notable disparities in the rates of response to EGFR-TKIs therapies are observed across various clinical subgroups of NSCLC patients, suggesting that the types of mutations, the occurrence of compound mutations, and additional clinical characteristics may exert an influence on treatment outcomes (4, 5). Malignant pleural effusion (MPE) represents a common complication in the advanced stages of NSCLC, with an estimated 50% of lung cancer patients experiencing MPE in the later stages, particularly those with lung adenocarcinoma (6). MPE significantly influences patients’ quality of life and survival rates. Over recent years, treatment strategies for NSCLC with MPE have been continuously updated, including traditional thoracentesis, pleurodesis, and intrapleural chemotherapy (7). Talc pleurodesis, a frequently utilized intervention for managing MPE, is supported by the National Comprehensive Cancer Network (NCCN) guidelines for addressing pleural effusion in patients who are not surgical candidates (8). Nonetheless, its specific use for MPE treatment has yet to receive official sanction in China. In clinical practice, many major oncology centers in China frequently recommend intrapleural chemotherapy with agents such as cisplatin or bleomycin. Although these therapeutic approaches can provide some degree of symptomatic relief for patients, the recurrence rate is still as elevated as 50% (9), leading to an unfavorable outcome for MPE sufferers, with a mere 6.4% five-year survival rate (10).

Currently, there is a scarcity of comparative research examining the efficacy and prognosis of EGFR-TKIs, either alone or in conjunction with intrapleural chemotherapy, in NSCLC patients, and it is not evident if the integration of EGFR-TKIs with intrapleural chemotherapy can markedly enhance the effectiveness of targeted treatment and patient prognosis. To explore this further, we initiated a real-world retrospective analysis involving 169 patients diagnosed with stage IVA-IVB NSCLC who exhibited EGFR mutations and had malignant pleural effusion at initial diagnosis. This study first compares the efficacy and prognosis of intrathoracic chemotherapy (ICT) plus EGFR-TKIs versus EGFR-TKI monotherapy in EGFR-mutated NSCLC patients with malignant pleural effusion, then employs multivariable analysis to identify independent prognostic factors that guide treatment selection.

## 2 Clinical data and methods

### 2.1 Clinical data

**Figure 1:**
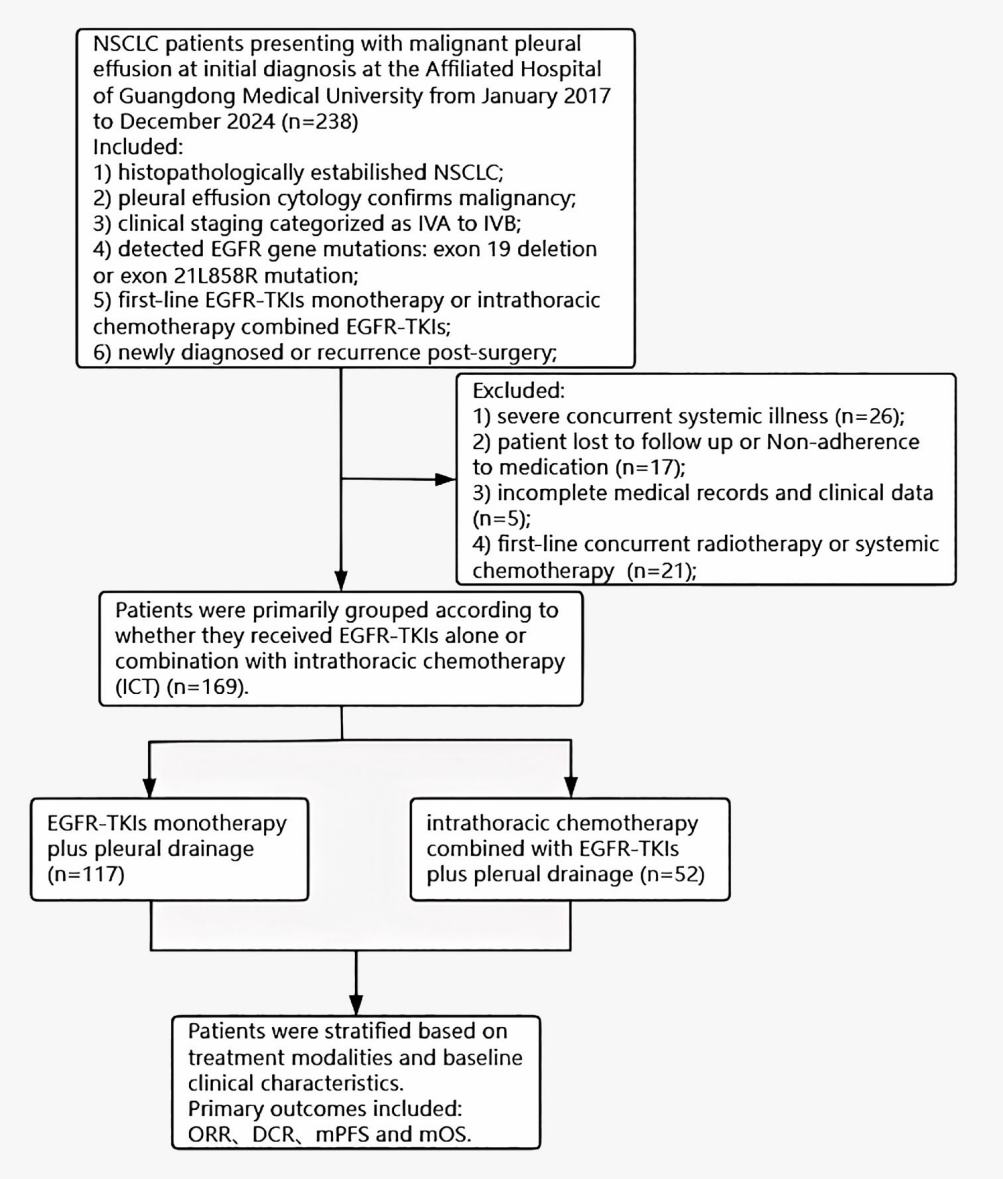
Flowchart of the study process. NSCLC, non-small cell lung cancer; EGFR, epidermal growth factor receptor; TKIs, Tyrosine kinase inhibitors; NSCLC, non-small cell lung cancer; ORR, objective response rate; DCR, disease control rate; mOS, median overall survival.

Consecutive patients with newly diagnosed stage IVA–IVB NSCLC and cytologically confirmed malignant pleural effusion who attended the Affiliated Hospital of Guangdong Medical University between January 2017 and December 2024 were screened (n = 238). Each patient had a pathological diagnosis of adenocarcinoma, squamous cell carcinoma or adenosquamous carcinoma and had commenced treatment with EGFR-TKIs. The standards required for recruitment in this study were as follows: 1) histopathologically established NSCLC; 2) pleural effusion cytology confirms malignancy; 3) clinical staging categorized as IVA to IVB; 4) detected EGFR gene mutations: exon 19 deletion or exon 21L858R mutation, included compound mutation; 5) first-line EGFR-TKIs monotherapy or intrathoracic chemotherapy combined EGFR-TKIs; 6) patients were either newly diagnosed or experienced a recurrence post-surgery; Eligible patients had either *de-novo* stage IVA–IVB disease or recurrent disease after curative-intent surgery, provided that malignant pleural effusion was documented at study entry and re-staged as IVA–IVB per AJCC 8th edition criteria. Exclusion criteria were: 1) severe concurrent systemic illness; 2) patient lost to follow up or Non-adherence to medication; 3) incomplete medical records and clinical data; 4) first-line concurrent radiotherapy or systemic chemotherapy; The conditions identified as serious comorbid illnesses encompass the following: cardiac diseases (such as a history of myocardial infarction, heart failure, valvular heart disease, and severe cardiac arrhythmias), chronic obstructive pulmonary disease (COPD), diabetes mellitus, cerebrovascular or peripheral vascular diseases, chronic renal failure, hepatitis and/or liver cirrhosis, hypertension, and severe autoimmune diseases. After exclusions, 169 patients fulfilled all criteria and were enrolled. The schematic representation of the study’s technical process is depicted in **Figure 1**. This study adhered to the ethical principles set forth in the Declaration of Helsinki (2013), as revised and updated. The study was approved by the Institutional Ethics Committee of the Affiliated Hospital of Guangdong Medical University (Approval No. PJKT2024-070) and individual consent for this retrospective analysis was waived. All patients included in the study provided written informed consent for Intrathoracic Chemotherapy (ICT) prior to treatment.

### 2.2 Experimental methods

Individuals diagnosed with NSCLC and who had genetic testing were included in this study, and details of their genetic mutations were gathered. The genetic examination was conducted predominantly by next-generation sequencing (NGS) and the amplification refractory mutation system (ARMS) fluorescence polymerase chain reaction (PCR), utilizing formalin-fixed paraffin-embedded (FFPE) tissue sections as the samples for analysis. Genetic testing findings for all patients were provided by reputable institutes, such as AmoyDx (Amoy Diagnostics, Xiamen, China). The testing scope covered exons 18 to 21 of the epidermal growth factor receptor (EGFR) gene, incorporating particular mutations that included L858R mutation in exon 21, exon 19 deletion (19 Del), exon 20 insertion (20 Ins), T790M, G719X, S768I, and L681Q. Furthermore, hotspot mutations were identified in several genes, including anaplastic lymphoma kinase (ALK), KRAS, NRAS, ROS1, RET, BRAF, PIK3CA, and HER2.

### 2.3 Efficacy assessment

A month following the commencement of EGFR-TKI targeted therapy, the first efficacy assessment was conducted. For patients with stable disease or a treatment response, a computerized tomography (CT) scan and other pertinent imaging studies were conducted bi-monthly to consistently assess the disease status. The effectiveness of the treatment was assessed applying the Response Evaluation Criteria in Solid Tumours (RECIST) version 1.1, classifying the outcomes into complete response (CR), partial response (PR), stable disease (SD), and progressing disease (PD). The objective response rate (ORR) was established using the calculation (CR + PR)/(CR + PR + SD + PD) × 100%, whereas the disease control rate (DCR) was computed with the equation (CR + PR + SD)/(CR + PR + SD + PD) × 100%.

### 2.4 Follow-up and research endpoints

Patient follow-up in this study was executed by the combination of outpatient records, inpatient medical records, and telephone follow-ups, with the end follow-up date established as June 7, 2025. Progression-free survival (PFS) was established as the period from commencing of EGFR-TKI targeted therapy to disease progression, mortality from any cause, or the follow-up deadline date (June 7, 2025). Overall survival (OS) was defined as the period from commencing of EGFR-TKI targeted therapy to death or the follow-up deadline date (June 7, 2025). Throughout the follow-up period, fatalities associated with NSCLC were documented as events, whereas data from patients who exhibited no disease progression or were alive at the completion of the follow-up were classified as censored data for the computation of cumulative survival rates.

### 2.5 Statistical methods

The examination of the statistical data for this research was conducted using IBM SPSS Statistics 27.0 software. A chi-square test has been applied for contrasting categorical data concerning clinical features and treatment outcomes. In terms of survival prognosis analysis, Kaplan-Meier survival analysis used the procedure estimate to estimate survival data, while the Cox proportional hazards regression model was exploited to evaluate factors affecting survival prognosis. Kaplan-Meier survival curves were produced with Prism software, additionally forest plots for the Cox proportional hazards model were constructed using RStudio software. A P-value below 0.05 was recognized as the criterion for statistical significance in this investigation.

## 3 Results

### 3.1 Clinical characteristics from participating patients

This study pertinent 169 patients with NSCLC diagnosed with MPE. The median age of those receiving therapy was 67 years, and all patients underwent treatment with EGFR-TKIs. Among them, 117 patients underwent pleural effusion drainage and received only TKI therapy, while 52 patients received TKI therapy, pleural effusion drainage, and ≥2 cycles of intrapleural chemotherapy. Among the enrolled patients, oral EGFR tyrosine-kinase inhibitors were continued until radiographic progression, unacceptable toxicity, or death. First-generation agents comprised gefitinib 250 mg once daily, erlotinib 150 mg once daily, or icotinib 125 mg three times daily (every 8 h); second-generation agents comprised afatinib 40 mg once daily or dacomitinib 45 mg once daily; and third-generation agents comprised osimertinib 80 mg once daily, almonertinib 110 mg once daily, or furmonertinib 160 mg once daily. Intrathoracic chemotherapy comprised three regimens: Cisplatin 30-60 mg intrapleurally on Days 1 and 3 (one cycle), for 2-4 cycles; Cisplatin 30-60 mg plus endostatin 45-90 mg intrapleurally on Days 1 and 3 (one cycle), for 2-4 cycles; Cisplatin 30-60 mg plus bevacizumab 100-300 mg intrapleurally on Days 1 and 3 (one cycle), for 2-4 cycles.

In this study, 22 patients (13.0%) were found to have compound mutations, that is, mutations at other gene loci in addition to the EGFR mutation. Compound mutations included TP53 (n=11), MET amplification (n=2), and various co-mutations involving TP53, ALK, ROS1, and others (n=9). All patients had their pleural effusion confirmed by 16-slice or 64-slice spiral CT scan at the time of diagnosis. Pleural effusion was categorized into three classifications according to the maximum depth observed on the CT cross-section: minimal (maximum depth ≤2cm), moderate (maximum depth >2cm and <1/2 of the thoracic cavity), and massive (maximum depth ≥1/2 of the thoracic cavity).

The chi-square test was employed to assess their distribution of clinical variables across the two treatment groups, revealing a balanced distribution that ensures the credibility of the study findings. **Table 1**. exhibits comprehensive review of the baseline characteristics of patients in the TKI monotherapy group and the combination therapy group.

**Table 1:** Contrast of clinical factors among cohorts with both treatment modalities.

Factors	Total (n = 169)	EGFR-TKIs monotherapy (n = 117)	ICT combined EGFR-TKIs (n = 52)	P
Gender, n(%)				0.96
Female	85 (50.30)	59 (50.43)	26 (50.00)	
Male	84 (49.70)	58 (49.57)	26 (50.00)	
Median age at diagnosis (years)				0.78
< 65	72 (42.60)	49 (41.88)	23 (44.23)	
≥ 65	97 (57.40)	68 (58.12)	29 (55.77)	
Smoking history, n(%)				0.23
No	137 (81.07)	92 (78.63)	45 (86.54)	
Yes	32 (18.93)	25 (21.37)	7 (13.46)	
Performance status, n(%)				0.24
0–1	116 (68.64)	77 (65.81)	39 (75.00)	
>1	53 (31.36)	40 (34.19)	13 (25.00)	
Pleural effusion volume, n(%)				0.26
Mild-moderate	108 (63.91)	78 (66.67)	30 (57.69)	
Large	61 (36.09)	39 (33.33)	22 (42.31)	
Clinical stage, n(%)				0.74
IVA	91 (53.85)	64 (54.70)	27 (51.92)	
IVB	78 (46.15)	53 (45.30)	25 (48.08)	
Generation of EGFR-TKIs, n(%)				0.88
1^st^ or 2^nd^ generation EGFR-TKIs	86 (50.89)	60 (51.28)	26 (50.00)	
3^rd^ generation EGFR-TKIs	83 (49.11)	57 (48.72)	26 (50.00)	
EGFR mutation status, n(%)				0.78
Exon 19 deletion	84 (49.70)	59 (50.43)	25 (48.08)	
21L858R mutation	85 (50.30)	58 (49.57)	27 (51.92)	
Compound mutations status, n(%)				0.27
No	147 (86.98)	104 (88.89)	43 (82.69)	
Yes	22 (13.02)	13 (11.11)	9 (17.31)	
Adrenal metastases, n(%)				0.61
No	134 (79.29)	94 (80.34)	40 (76.92)	
Yes	35 (20.71)	23 (19.66)	12 (23.08)	
Bone metastases, n(%)				0.38
No	93 (55.03)	67 (57.26)	26 (50.00)	
Yes	76 (44.97)	50 (42.74)	26 (50.00)	
Liver metastases, n(%)				0.88
No	128 (75.74)	89 (76.07)	39 (75.00)	
Yes	41 (24.26)	28 (23.93)	13 (25.00)	
Brain metastases, n(%)				0.82
No	135 (79.88)	94 (80.34)	41 (78.85)	
Yes	34 (20.12)	23 (19.66)	11 (21.15)	

EGFR, epidermal growth factor receptor; TKIs, Tyrosine kinase inhibitors; ICT, Intrathoracic chemotherapy.

### 3.2 Efficacy response

This clinical study comprised 169 patients undergoing EGFR-TKI treatment, of whom 86 attained a PR, resulting in an overall ORR of 50.1% (86/169) while a DCR of 85.8% (145/169).

Regarding the objective response rate (ORR), no statistically significant difference was detected between the EGFR-TKI monotherapy cohort and the cohort receiving thoracic perfusion chemotherapy in conjunction with EGFR-TKIs (50.4% vs. 51.9%, p = 0.86). Patients exhibiting a performance status (PS) score of 0-1 demonstrated a superior ORR in contrast to those with a PS score exceeding 1 (56.9% vs. 37.7%, p = 0.021). Patients administered third-generation EGFR-TKIs exhibited a considerably superior overall response rate (ORR) in contrast to others treated with first- or second-generation EGFR-TKIs (61.4% vs. 40.7%, p = 0.007). The ORR was substantially greater in patients with exon 19 deletion mutations than in those with L858R mutations (58.8% vs. 42.9%, p = 0.038). Similarly, Patients without compound mutations presented an outstanding ORR compared to those with compound mutations (55.1% vs. 22.7%, p = 0.005). Patients without hepatic metastasis also had a more significant ORR in contrast to individuals with hepatic metastasis (56.3% vs. 34.1%, p = 0.014).

Regarding DCR, no statistically significant difference was discovered between the EGFR-TKI monotherapy cohort and the group receiving thoracic perfusion chemotherapy in conjunction with EGFR-TKIs (84.6% vs. 88.5%, p = 0.51). The patients with a PS score of 0 - 1 exhibited a substantially higher DCR compared to the individuals with a PS level exceeding 1 (90.5% vs. 75.5%, p = 0.009). Patients administered third-generation EGFR-TKIs exhibited a markedly superior DCR in contrast to individuals treated with first- or second-generation EGFR-TKIs (94.0% vs. 77.9%, p = 0.003). Patients devoid of hepatic metastasis exhibited a markedly superior DCR in contrast to individuals with hepatic metastasis (89.1% vs. 75.6%, p = 0.032). The assessments of the efficacy evaluation for each clinical characteristic subgroup are presented in **Table 2**.

**Table 2:** Evaluation of ORR and DCR for EGFR-TKIs across several clinical features and treatment method categories.

Features	n	Objective response rate				Disease control rate			
		n	Rate (%)	χ²	p	n	Rate (%)	χ²	p
Overall Rate	169	86	50.1			145	85.8		
Gender									0.073
Female	85	48	56.5			77	90.6		
Male	84	38	45.2	2.13	0.14	68	81	3.22	0.073
Median age at diagnosis (years)									
< 65	72	36	50			63	87.5		
≥ 65	97	50	51.5	0.04	0.84	82	84.5	0.3	0.59
Smoking history									
No	137	68	49.6			117	85.4		
Yes	32	18	56.3	0.45	0.50	28	87.5	0	0.98
Performance status									
0–1	116	66	56.9			105	90.5		
≥ 2	53	20	37.7	5.34	0.021	40	75.5	6.76	0.009
Pleural effusion volume									
Mild-moderate	108	51	47.2			91	84.3		
Large	61	35	57.4	1.61	0.21	54	88.5	0.58	0.45
Clinical stage									
IVA	91	52	57.1			81	89		
IVB	78	34	43.6	3.09	0.079	64	82.1	1.67	0.2
Generation of EGFR-TKIs									
1^st^ or 2^nd^ generation EGFR-TKIs	86	35	40.7			67	77.9		
3^rd^ generation EGFR-TKIs	83	51	61.4	7.28	0.007	78	94.0	8.95	0.003
EGFR mutation status									
Exon 19 deletion	85	50	58.8			77	90.6		
21L858R mutation	84	36	42.9	4.31	0.038	68	81.0	3.22	0.073
Compound mutations status									
No	147	81	55.1			126	85.7		
Yes	22	5	22.7	8.03	0.005	19	86.4	0	1.000
Adrenal metastases									
No	134	73	54.5			118	88.1		
Yes	35	13	37.1	3.34	0.068	27	77.1	1.89	0.17
Bone metastases									
No	93	50	53.8			84	90.3		
Yes	76	36	47.4	0.68	0.41	61	80.3	3.47	0.062
Liver metastases									
No	128	72	56.3			114	89.1		
Yes	41	14	34.1	6.07	0.014	31	75.6	4.61	0.032
Brain metastases									
No	135	69	51.1			117	86.7		
Yes	34	17	50	0.01	0.91	28	82.4	0.14	0.71
Treatment modality									
EGFR-TKIs monotherapy	117	59	50.4			99	84.6		
ICT combined EGFR-TKIs	52	27	51.9	0.03	0.86	46	88.5	0.44	0.51

ORR, objective response rate; DCR, disease control rate; EGFR, epidermal growth factor receptor; TKIs, Tyrosine kinase inhibitors; ICT, Intrathoracic chemotherapy.

### 3.3 Survival analysis

The median progression-free survival (mPFS) and median overall survival (mOS) for the 169 individuals in the current investigation were 13.1 months and 19.6 months, respectively. Among the median follow-up period of 37.5 months, progression of the illness was observed in 67.5% (114/169) of patients, together with 56.2% (95/169) of patients succumbed to the disease. No fatalities associated with the treatment were documented.

**Figure 2:**
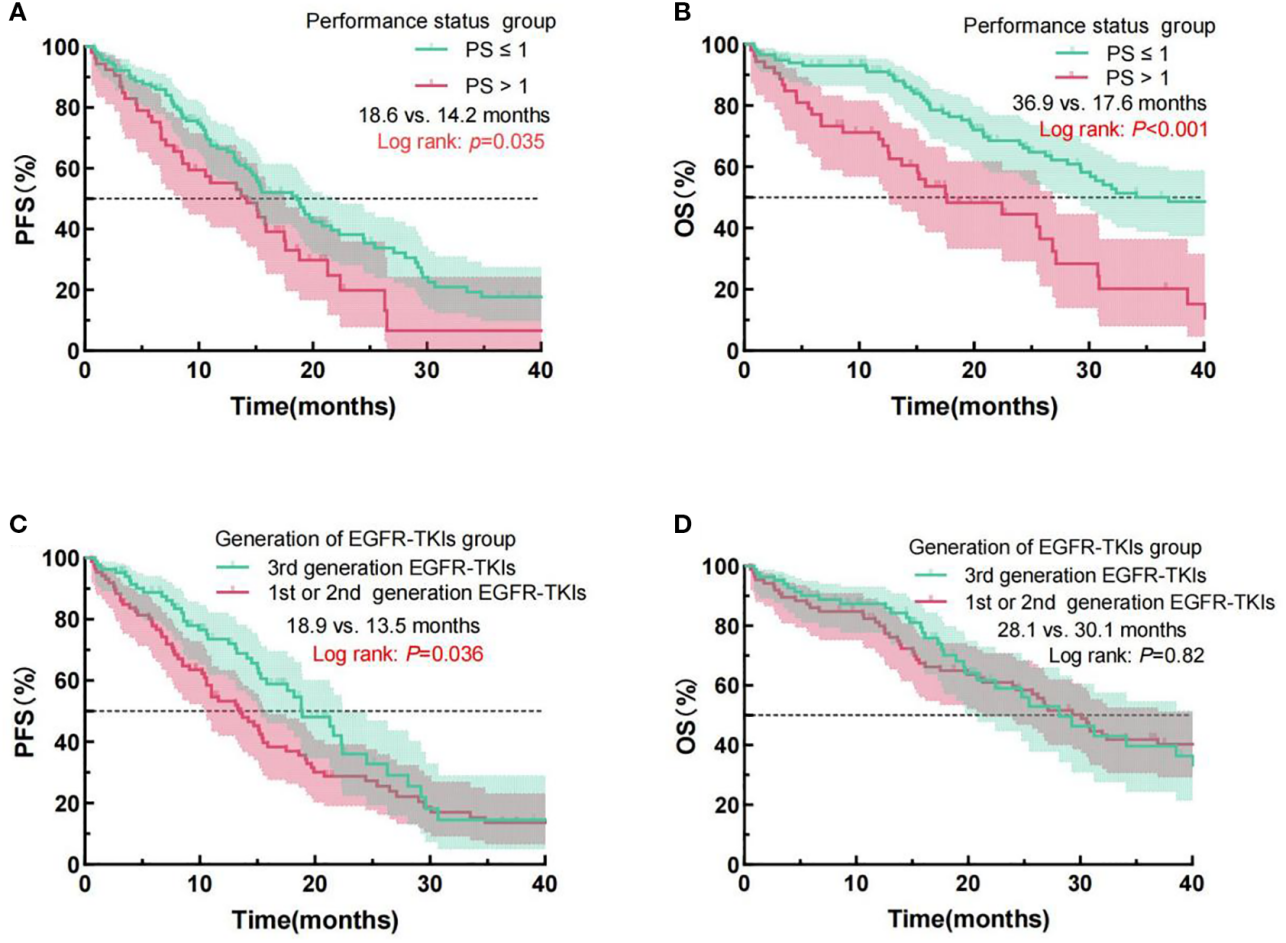
Examination of PFS and OS curves for NSCLC patients administered EGFR-TKIs, classified by PS **(A, B)** and EGFR-TKI generation **(C, D)**. PFS, progression-free survival; OS, overall survival; NSCLC, Non-small cell lung cancer; PS, performance status; EGFR, epidermal growth factor receptor; TKIs, Tyrosine kinase inhibitors.

The Kaplan-Meier survival investigation revealed the individuals with a performance status score of 0-1 exhibited a markedly extended mPFS of 18.6 months, compared to 14.2 months for individuals with a PS score exceeding 1 (Log Rank p = 0.035), as well as a mOS of 36.9 months versus 17.6 months (Log Rank p < 0.001). Moreover, patients administered third-generation EGFR-TKIs had a significantly extended mPFS of 18.9 months, in contrast to 13.5 months for individuals treated with first- or second-generation EGFR-TKIs (Log Rank p = 0.036). No statistically significant difference in mOS was reported (28.1 months vs. 30.1 months, Log Rank p = 0.82). **Figure 2** exhibits the prognostic analysis outcomes for PFS and OS depending upon these two clinical characteristics.

**Figure 3:**
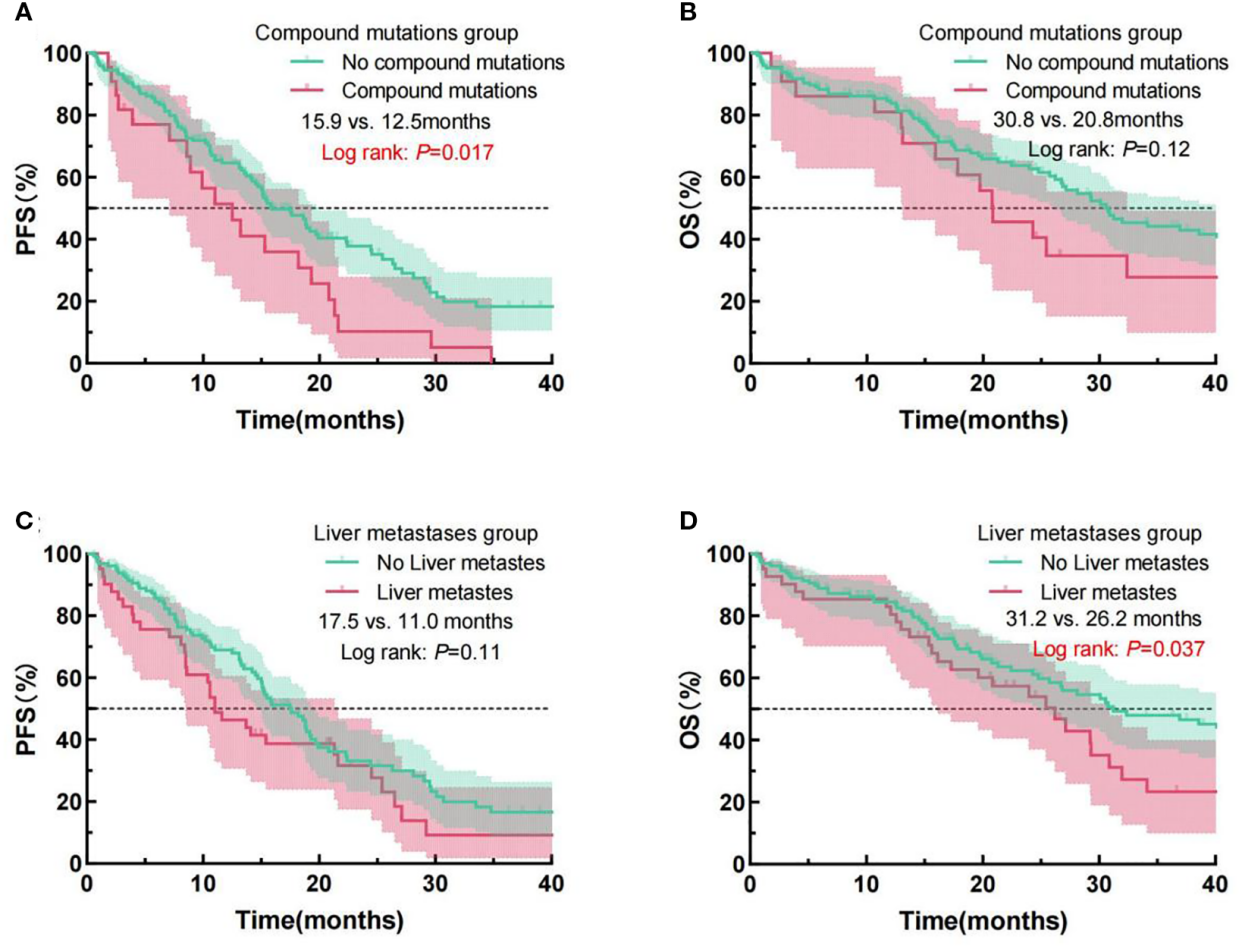
Examination of PFS and OS curves for NSCLC patients administered EGFR-TKIs, classified by compound mutations status **(A, B)**, and the presence of liver metastases **(C, D)**. PFS, progression-free survival; OS, overall survival; NSCLC, Non-small cell lung cancer; PS, performance status; EGFR, epidermal growth factor receptor; TKIs, Tyrosine kinase inhibitors.

Additionally, we conducted prognostic analyses of PFS and OS for patients categorized by compound mutation status and liver metastasis status (see **Figure 3**). The findings indicated individuals who lacking compound mutations exhibited a substantially extended mPFS (15.9 months vs. 12.5 months, Log Rank p = 0.017) compared with those with compound mutations. Regardless of the compound mutation group not exhibiting a statistically noteworthy distinction in mOS (30.8 months vs. 20.8 months, Log Rank p = 0.12), the prognosis curve of the no compound mutation group was consistently above that of the compound mutation group, with a trend of separation over time. Patients without liver metastasis exhibited a longer mOS (31.2 months vs. 26.2 months, Log Rank p = 0.037) in comparison to those with liver metastasis. However, no difference in significance was detected in mPFS (17.5 months vs. 11.0 months, Log Rank p = 0.11).

**Figure 4:**
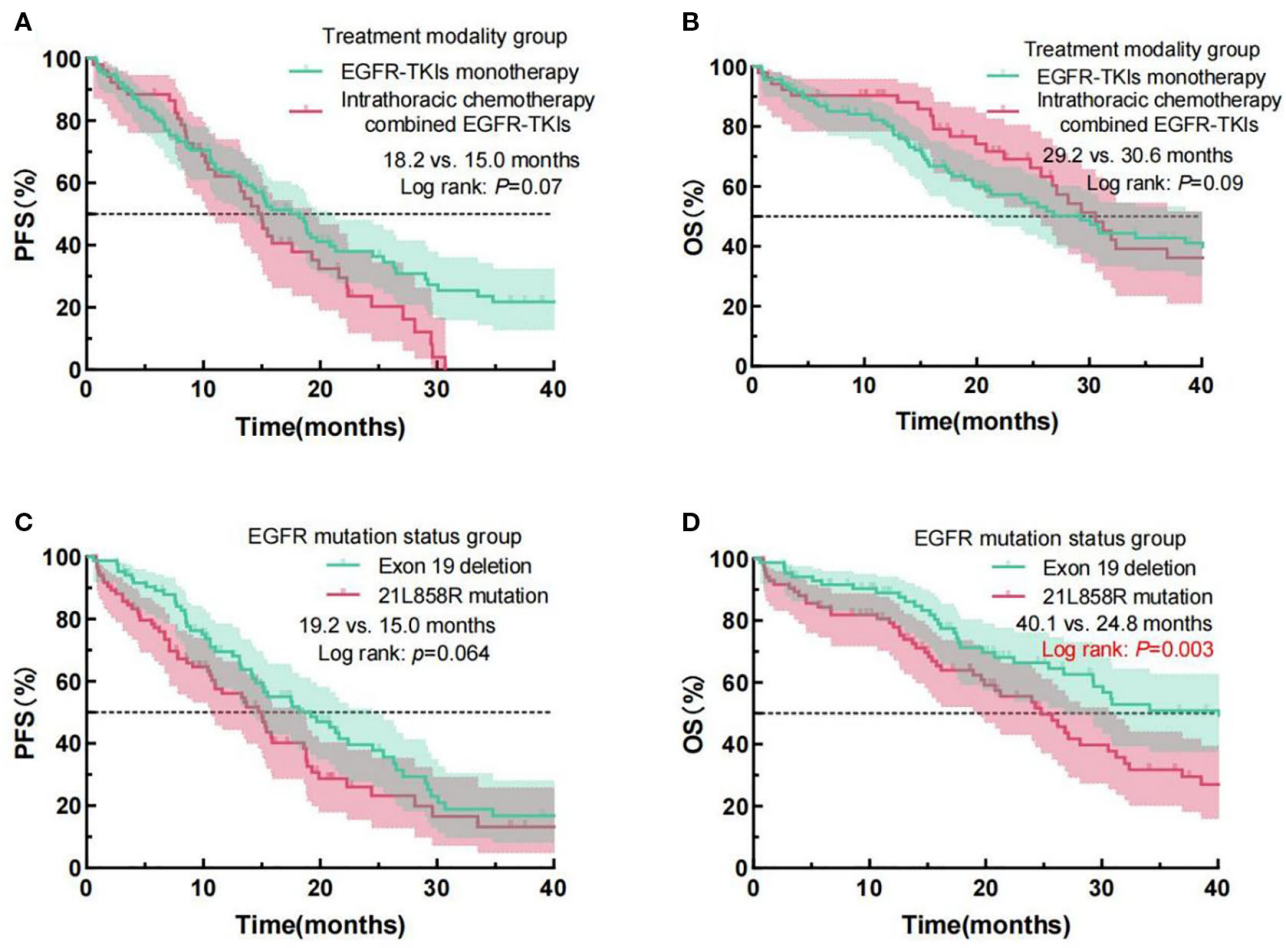
Examination of PFS and OS curves for NSCLC patients administered EGFR-TKIs, classified by different treatment modality groups **(A, B)** and EGFR mutation status **(C, D)**. PFS, progression-free survival; OS, overall survival; NSCLC, Non-small cell lung cancer; PS, performance status; EGFR, epidermal growth factor receptor; TKIs, Tyrosine kinase inhibitors.

Kaplan-Meier survival analysis revealed no significantly different outcomes in mPFS (18.2 months vs. 15.0 months, Log Rank p = 0.07) or mOS (29.2 months vs. 30.6 months, Log Rank p = 0.09) between EGFR-TKI monotherapy and thoracic perfusion chemotherapy combined with EGFR-TKIs. In the comparison between L858R mutations and exon 19 deletion, individuals with the exon 19 deletion exhibited a significantly prolonged mOS (40.1 months vs. 24.8 months, Log Rank p = 0.003) compared to those with the L858R mutation. Despite the absence of a significant variance in mPFS (19.2 months vs. 15.0 months, Log Rank p = 0.064) between the mutation groups, the survival curve for the exon 19 deletion group was consistently above that of the L858R mutation group, with a trend of separation over time. The prognostic analysis results for PFS and OS according to these two clinical characteristics are presented in **Figure 4**. The Kaplan-Meier analysis results for PFS and OS across all clinical variables are detailed in **Table 3**.

**Table 3:** Survival analysis of PFS and OS across various clinical features and different therapy modality groups.

Factors	PFS (months)	Log rank P	OS (months)	Log rank P
Gender		0.15		0.15
Female	18.8		32.4	
Male	14.1		25.4	
Median age at diagnosis		0.55		0.26
< 65	17.5		30.6	
≥ 65	15.4		27.1	
Smoking history		0.67		0.93
No	15.4		29.3	
Yes	18.2		30.6	
Performance status		0.035		<0.001
0–1	18.6		36.9	
> 1	14.2		17.6	
Pleural effusion volume		0.79		0.88
Mild-moderate	15.3		29.3	
Large	17.5		30.6	
Clinical stage		0.86		0.31
IVA	17.5		30.1	
IVB	14.1		26.2	
Generation of EGFR-TKIs		0.036		0.82
1^st^ or 2^nd^ generation EGFR-TKIs	13.5		30.1	
3^rd^ generation EGFR-TKIs	18.9		28.1	
EGFR mutation status		0.064		0.003
Exon 19 deletion	19.2		40.1	
21L858R mutation	15.0		24.8	
Compound mutations status		0.017		0.12
No	15.9		30.8	
Yes	12.5		20.8	
Adrenal metastases		0.054		0.54
No	15.9		30.6	
Yes	10.6		24.3	
Bone metastases		0.13		0.22
No	18.8		32.4	
Yes	14.1		25.7	
Liver metastases		0.11		0.037
No	17.5		31.2	
Yes	11.0		26.2	
Brain metastases		0.71		0.84
No	15.9		29.3	
Yes	13.7		27.1	
Treatment modalities		0.07		0.09
ICT combined EGFR-TKIs	15.0		30.6	
EGFR-TKIs monotherapy	18.2		29.2	

PFS, progression-free survival; OS, overall survival; EGFR, epidermal growth factor receptor; TKIs, Tyrosine kinase inhibitors; ICT, Intrathoracic chemotherapy.

### 3.4 Univariate and multivariate analysis

This study incorporated all clinical variables and the two treatment modes in the univariate analysis. Variables with a P-value less than 0.2 in the univariate evaluation were deemed of statistical significance and incorporated into the multivariate analysis. The univariate analysis results indicated that the variables correlated with PFS comprised: gender, performance status, generation of EGFR-TKIs, treatment modality, EGFR mutation status, compound mutations status, adrenal metastases, bone metastases, and liver metastases. The variables associated with OS included: gender, performance status, EGFR mutation status, compound mutations status, and liver metastases.

**Figure 5:**
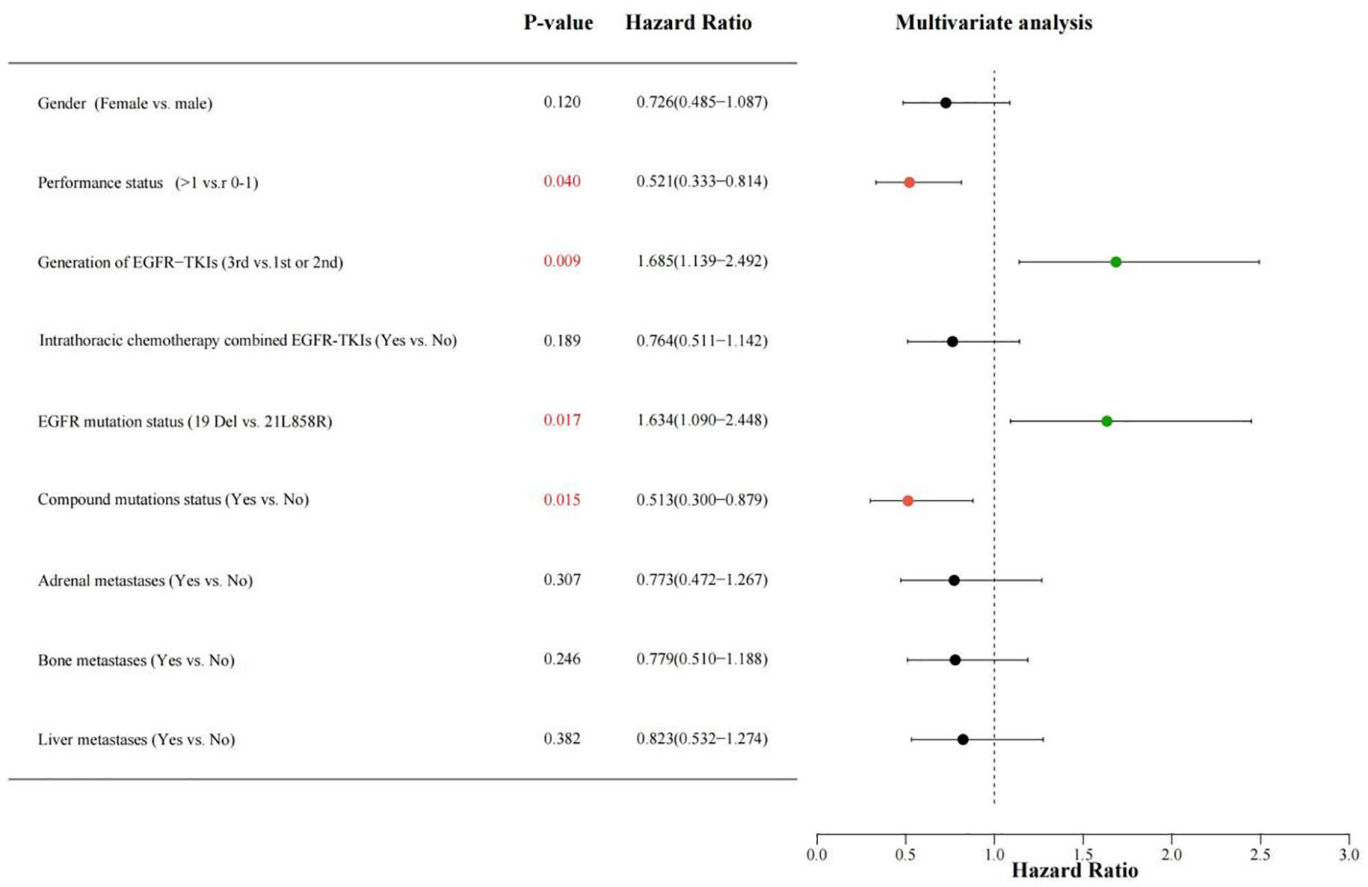
Multiple-variable COX regression evaluation investigating the correlation among different classification factors such as gender (Female vs. male), PS score (> 1 vs. 0-1), generation of EGFR-TKIs (1st and 2nd,or 3rd), intrathoracic chemotherapy combined EGFR-TKIs(Yes vs.No), EGFR mutation status (Exon 19 deletion vs. 21L858R), compound mutations status(Yes vs.No), Adrenal metastases (Yes vs.No), bone metastases(Yes vs.No), liver metastases (Yes vs. No), with PFS for patients receiving EGFR-TKIs. A HR greater than 1 suggests a beneficial predictor for PFS, while an HR less than 1 denotes an adverse predictor for PFS. PS, performance status; EGFR, epidermal growth factor receptor; TKIs, Tyrosine kinase inhibitors; PFS, progression-free survival; HR, hazard ratio.

**Figure 6:**
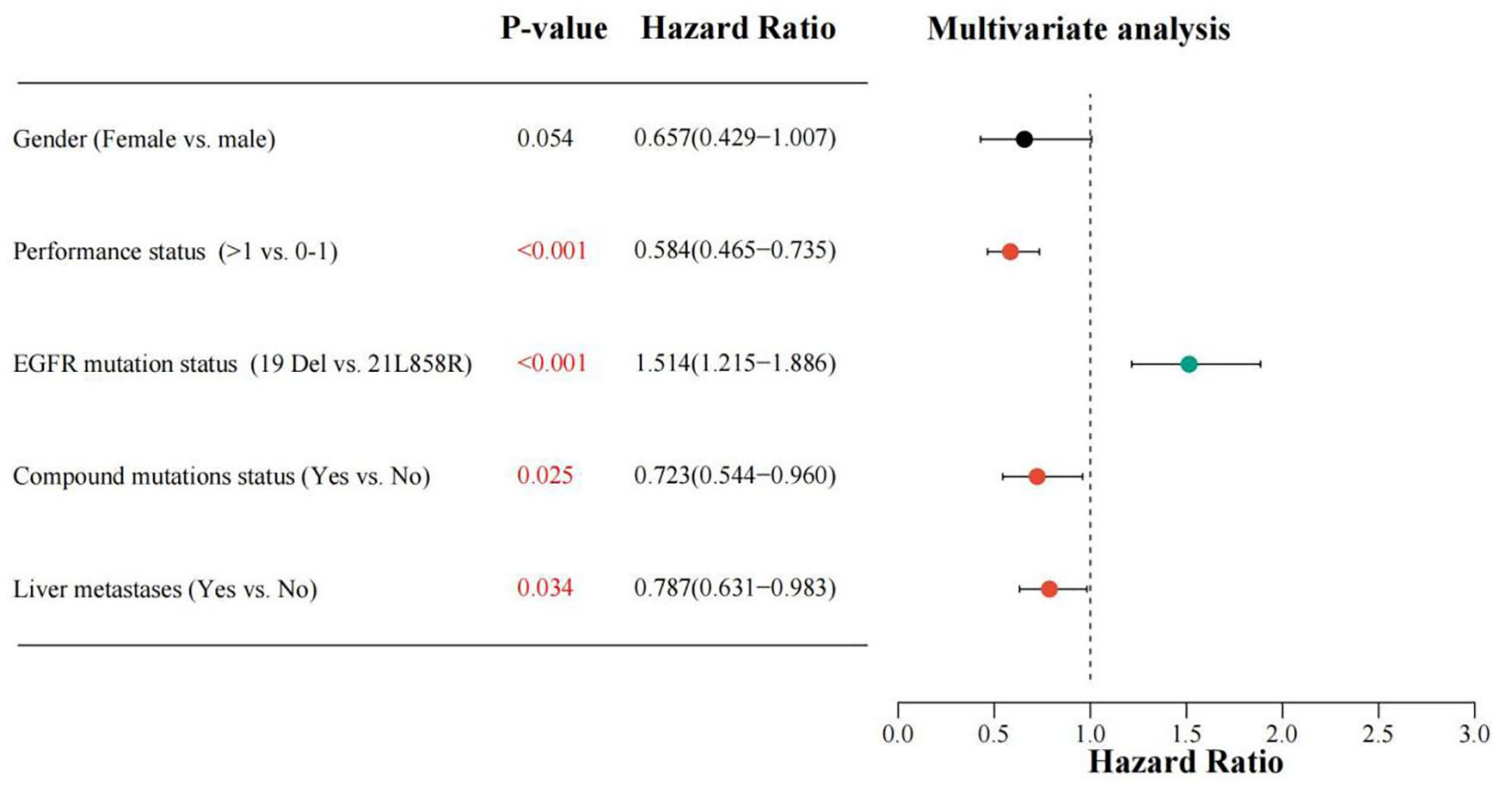
Multiple-variable COX regression evaluation investigating the correlation among different classification factors such as gender (Female vs. male), PS score (> 1 vs. 0-1), EGFR mutation status (Exon 19 deletion vs. 21L858R), compound mutations status(Yes vs.No), liver metastases (Yes vs. No), with OS for patients receiving EGFR-TKIs. A HR greater than 1 suggests a beneficial predictor for OS, while an HR less than 1 denotes an adverse predictor for OS. PS, performance status; EGFR, epidermal growth factor receptor; TKIs, Tyrosine kinase inhibitors; OS, overall survival; HR, hazard ratio.

After incorporating the aforementioned variables into the multivariate analysis, the results demonstrated that performance status, generation of EGFR-TKIs, EGFR mutation status, and compound mutations status were independently predictive variables for PFS in patients with EGFR mutation-positive NSCLC complicated by MPE receiving first-line EGFR-TKIs treatment (see **Figure 5**). Performance status, EGFR mutation status, compound mutations status, and liver metastases were independently predictive variables for OS in patients (see **Figure 6**).

Ultimately, we identified the following factors as having an impact on patient prognosis: the utilization of third-generation EGFR-TKIs and the presence of the 19Del mutation were favorable factors for PFS, while a PS score >1 and the presence of compound mutations were unfavorable factors for PFS. Similarly, the 19Del mutation was a favorable factor for OS, whereas a PS score >1, the presence of compound mutations, along with liver metastases were detrimental variables for OS.

## 4 Discussion

MPE is a prevalent complication in individuals with advanced lung cancer, adversely affecting their quality of life and prognosis (11). In clinical practice, intrapleural administration of chemotherapeutic agents or biological agents is a commonly used method to control MPE (12). Nevertheless, research on malignant pleural effusion and intrapleural treatment in patients with EGFR mutation-positive non-small cell lung cancer is rather limited. At present, there is not sufficient evidence to clarify whether intrapleural chemotherapy combined with EGFR-TKIs can substantially enhance the effectiveness of targeted therapy and the survival outcome of patients in comparison to EGFR-TKIs monotherapy.

This study’s findings indicate that, among patients with EGFR-mutated NSCLC complicated by malignant pleural effusion, there existed no significant variations in ORR (50.4% vs. 51.9%, Log Rank p=0.86), DCR (84.6% vs. 88.5%, Log Rank p=0.51), mPFS (18.2 months vs. 15.0 months, Log Rank p=0.07), and mOS (29.2 months vs. 30.6 months, Log Rank p=0.09) between the EGFR-TKI monotherapy cohort and the intrathoracic chemotherapy combined with EGFR-TKI cohort. Based on these findings, this study concludes that, in this patient population, the combined use of intrathoracic chemotherapy and EGFR-TKI fails to substantially augment the efficacy of targeted therapy or improve survival prognosis in comparison with EGFR-TKI monotherapy alone. In the study by Wang et al., a comparison was made regarding the EGFR-TKI monotherapy cohort and the cohort receiving combined TKI and intrathoracic treatment. The results showed no significant statistical differences in intrathoracic ORR (65.5% vs. 58.1%, Log Rank p=0.45), mPFS (10.3 months vs. 9.9 months, Log Rank p=0.75), and median intrapleural progression-free survival (iPFS) (11.9 months vs. 12.7 months, Log Rank p=0.65). However, the study had a restricted number of patients (n=101) and failed to account for additional clinical features or potential confounding variables that could influence the results (13). A study examining the recurrence rate of pleural effusion regarding gefitinib monotherapy and gefitinib in conjunction with pleurodesis (utilizing minocycline or OK432) revealed an insignificant difference in iPFS between the non-pleurodesis group (39 patients) and the pleurodesis group (17 patients) (5.0 months vs. 4.8 months, Log Rank p=0.81). This suggests that gefitinib monotherapy is equally effective in controlling pleural effusion compared with gefitinib combined with pleurodesis (14). Verma et al. conducted a study comparing the effectiveness of EGFR-TKI monotherapy to that of EGFR-TKI in conjunction with talc pleurodesis in patients suffering from advanced lung adenocarcinoma and malignant pleural effusion possessing EGFR-activating mutations. In the cohort of 39 patients, EGFR-TKI monotherapy showed a trend toward longer MPE recurrence-free survival compared with the combination group (median 352 days vs. 298 days; P=0.59) (15). In alignment with the conclusions of the current research, these investigations indicate that for patients with EGFR-mutated NSCLC complicated by MPE, intrapleural chemotherapy or pleurodesis may not confer additional benefits regarding PFS and prevention of MPE recurrence. Recently, a propensity-score matched trial conducted by Yang et al. indicated that intrathoracic perfusion therapy may be correlated individually with OS (HR, 0.557; 95% CI, 0.327-0.951; P = 0.03). Nevertheless, this study did not conduct further survival analysis between the two groups, nor did it include variables such as metastatic organs and co - mutations that might affect OS. The model constructed in this study had a significant selection bias (16).

Previous key clinical trials, such as FLAURA (17), FURLONG (18), and AENEAS (19), have demonstrated the third-generation EGFR-TKIs provide enhanced PFS and OS relative to first-generation drugs in all EGFR-mutated NSCLC patients, particularly in those with cerebral metastases. Our research additionally shown that third- generation EGFR-TKIs have a distinct PFS advantage over first- and second- generation drugs in EGFR-mutated NSCLC patients with MPE. Both the British Thoracic Society (BTS) Pleural Disease Guideline 2023 and the ATS/STS/STR Clinical Practice Guideline 2018 advocate for thoracic drainage to promptly alleviate dyspnea and enhance the quality of life in patients with symptomatic MPE, followed by systemic treatment initiation (20, 21). All 169 patients enrolled in this study received thoracic drainage per guideline recommendations as part of their first-line therapy. Given that our study demonstrated no superior efficacy or survival benefits of intrathoracic chemotherapy combined with EGFR-TKIs compared to monotherapy with EGFR-TKIs, and considering that international clinical guidelines recommend pleural drainage as a standard approach for managing malignant pleural effusion (MPE) and the significant efficacy of third-generation EGFR-TKIs observed in our study for treating EGFR mutation-positive lung cancer patients with MPE, we speculate that the combination of third-generation EGFR-TKIs and pleural drainage may be a more rational treatment option for this patient population. Future studies are needed to further validate this hypothesis.

When different generations of EGFR-TKIs are used in first-line therapy for NSCLC patients, there is compelling evidence that exon 19 deletion mutations confer greater clinical benefits to patients than exon 21 L858R mutations, a conclusion that has been confirmed in multiple studies (22–24). There are several potential mechanisms underlying the differences in treatment outcomes between these two classic mutations. From a molecular perspective, the exon 19 deletion mutation is situated in the N-lobe C-helix domain of the EGFR enzyme, near to the ATP-binding site, resulting in increased sensitivity to TKIs. In contrast, the L858R mutation in exon 21 is located in the loop that activates (A-loop) of the C-lobe of the EGFR kinase domain, distal to the ATP-binding site, potentially explaining its durability against TKIs (25). Moreover, compared with exon 19 deletion mutations, the proportion of compound mutations at other gene loci (e.g., 21-L861Q, 18-G719X, 20-Ins) is higher in patients with the L858R mutation, which may also be one of the main reasons for the differences in treatment outcomes (26–28). This view was preliminarily validated in our study, where 86.4% (19/22) of the 22 co-mutation patients had the L858R mutation. Recently studies have also explained the high prognostic benefits of targeted therapy for exon 19 deletion mutations from the perspective of EGFR gene mutation abundance or circulating tumor DNA (ctDNA) quantification. These studies have shown that the prevalence of exon 19 deletion mutations is greater in the high mutation abundance cohort, thus making it significantly better than the L858R mutation in terms of clinical prognosis (29–31).

In the entire population of patients with EGFR-mutated NSCLC, compound mutations have been confirmed as a prognostic factor in multiple studies, with the most common TP53 mutation attracting particular attention (27, 32, 33). Vokes et al. pointed out that tumors with TP53 mutations exhibit higher mutational burden and higher mutation rates after treatment and smoking, which are closely related to poor survival prognosis (34). Recently, Wei et al. discovered that patients with the L858R mutation who also possessed TP53 compound mutations experienced markedly reduced survival and accelerated development of tolerance to EGFR-TKI treatment compared to those without TP53 compound mutations. However, similar trends were not observed in patients with 19 Del mutations or other EGFR subgroups, indicating that TP53 compound mutations are an adverse prognostic factor specifically for L858R - mutated NSCLC patients (35). Among the 22 compound mutation patients included in our study, 68.2% (15/22) had TP53 mutations, and 86.4% (19/22) of the co-mutation patients had the L858R mutation. These findings align with existing research and further illustrate that compound mutations adversely affect survival prognosis in the cohort of EGFR-mutated NSCLC patients with MPE. Our investigation demonstrated that in EGFR-mutated NSCLC patients with MPE, compound mutations at additional loci alongside the EGFR mutation represent a prognostic risk factor for reduced PFS and OS. In our cohort of 22 patients harbouring co-occurring EGFR and non-EGFR alterations, only conventional EGFR-TKI monotherapy was prescribed; no matched targeted agents for the secondary drivers were used. Among the 22 patients with compound mutations, none received matched inhibitors against the secondary drivers. TP53 alterations remain pharmacologically orphan, whereas MET amplification and ALK/ROS1 fusions could have been targeted by crizotinib (36), capmatinib (37), or entrectinib (38). However, the present cohort exclusively comprised patients receiving EGFR-TKI monotherapy; enrolment of individuals scheduled for matched targeted agents against secondary driver alterations was not permitted. Consequently, this restriction may account for the uniformly inferior outcomes observed among patients harboring uncommon co-alterations.

Identifying organ metastases linked to the outcome of patients undergoing EGFR-TKI therapy is essential for educating patients and directing treatment decisions. Hepatic metastasis has previously been shown as an independent predictive factor for diminished OS after EGFR-TKI therapy in multiple prior investigations (39–41). This study indicates that patients with MPE and hepatic metastasis had significantly reduced ORR, DCR, and mOS compared to those without hepatic metastasis, corroborating previous findings. The study by Jiang et al. revealed that the hepatocyte growth factor (HGF)/MET signaling pathway may be one of the causes of the aforementioned phenomenon. In patients with hepatic metastasis from various malignancies, HGF levels are often elevated. The activation of c-MET by HGF could enhance ErbB3/PI3K/AKT signalling, which may heighten the tolerance to EGFR-TKIs in hepatic metastasis, resulting in a worse prognosis and diminished treatment efficacy (42). Chen et al. have also indicated that in the microenvironment of tumours of hepatic metastasis, its expression level of vascular endothelial growth factor (VEGF) is elevated compared with additional locations of metastases (43). VEGF not only enhances neoplasm angiogenesis but additionally stimulates the proliferation of cancer cells and influences the immune-suppressive network, consequently advancing the evolution of NSCLC (44–46). Recently, a study by You et al. found that combination anti - angiogenic therapy may confer greater OS prognostic benefits to patients with hepatic metastasis than to those without (47). However, data on the optimal systemic or local consolidative treatment for hepatic metastatic tumors are extremely limited (40). More clinical studies are needed in the future to explore the efficacy and survival benefits of combining EGFR-TKIs with systematic or local therapy strategies for individuals with hepatic metastatic lung cancer, to further the advancement of customized precision treatment.

Nonetheless, our research has some limitations. First, the data originated from a single-center database, which obviously limits the applicability of our results and requires validation through multicenter investigations. Second, our investigation was confined to evaluating the effects of EGFR-TKIs as monotherapy and intrapleural chemotherapy combined with EGFR-TKIs on the objective response of solid tumors and long-term prognosis in patients. We did not include assessments of pleural effusion control rates or other relevant metrics, which may have limited the comprehensive evaluation of the overall therapeutic efficacy. Another limitation of this study is that, focusing on first-line treatment in the NSCLC patient population, systematic collection of repeated genetic testing data following disease progression was not performed. This precludes detailed analysis of acquired resistance mechanisms (such as the emergence of T790M mutations or other relevant genetic alterations), which could have provided critical insights into treatment failure patterns in this cohort of EGFR-mutated NSCLC patients with malignant pleural effusion. This limitation underscores the necessity for future prospective studies incorporating longitudinal genetic testing to track dynamic changes in the genomic landscape upon disease progression, thereby enhancing understanding of resistance evolution in this specific population.

## 5 Conclusion

Despite our study failing to demonstrate superior efficacy or survival benefits of ICT combined with EGFR-TKIs compared to EGFR-TKI monotherapy, considering that international clinical guidelines recommend pleural drainage as a standard approach for managing MPE and the significant efficacy of third-generation EGFR-TKIs observed in our study for treating EGFR mutation-positive lung cancer patients with MPE, we speculate that the combination of third-generation EGFR-TKIs and pleural drainage may be a more rational treatment option for this patient population. Future studies are needed to further validate this hypothesis. In addition, when evaluating the potential benefits of EGFR-TKIs in this patient population, other independent prognostic factors should also be considered.

## Data Availability

The raw data supporting the conclusions of this article will be made available by the authors without undue reservation.
